# Fermentation Process Optimization for High 2-Phenylethanol Aroma Whisky

**DOI:** 10.3390/ijms27114759

**Published:** 2026-05-25

**Authors:** Kadireya Tuerxun, Zhuoling Ding, Xueqing Luo, Shishui Zhou

**Affiliations:** School of Biology and Biological Engineering, South China University of Technology, Guangzhou 510006, China; 19861550855@163.com (K.T.); zhuoling_ding@163.com (Z.D.); 15188283211@163.com (X.L.)

**Keywords:** *Saccharomyces cerevisiae*, 2-phenylethanol, CRISPR-Cas9, *ARO8* gene, fermentation process optimization

## Abstract

2-Phenylethanol (2-PE) is a key aromatic alcohol contributing to the rose-like odor in brewed wines, primarily synthesized by yeast metabolism with a typical yield of less than 100 mg/L. To enhance the 2-PE content in brewed wines, this study used CRISPR-Cas9 gene editing technology to delete the *ARO8* gene (encoding aromatic transaminase I) in *Saccharomyces cerevisiae* SY. The single-factor experiments were performed to optimize the fermentation process, and the 2-PE content in the brewed wine was measured by high-performance liquid chromatography. The results demonstrated that the 2-PE content in whisky fermented by the SY-A8 was 0.73 g/L, increasing 23.73% compared to SY. The fermentation conditions of SY-A8 were optimized through single-factor experiments and the Box–Behnken design. The optimal conditions were a sugar concentration of 46.30 g/L, a fermentation time of 6 days, and an L-phenylalanine concentration of 1.43 g/L. The high 2-phenylethanol aroma whisky was brewed with a higher 2-phenylethanol content of 3.68 g/L in a 1 L fermenter at the optimal conditions. In conclusion, the modification of *Saccharomyces cerevisiae* by CRISPR-Cas9 gene editing combined with fermentation process optimization provides an effective technical strategy for improving the 2-PE content in whisky, thereby providing a research perspective for the flavor enhancement of whisky and other brewed wines.

## 1. Introduction

2-phenylethanol (2-PE) is an aromatic alcohol with a characteristic rose-like odor that commonly exists in the essential oils of various plants, including rose and jasmine [1]. In addition to its role as a natural fragrance, 2-PE exhibits diverse biological activities. Studies have demonstrated that 2-PE possesses significant antimicrobial activity, effectively inhibiting the growth of Gram-negative bacteria, cocci, bacilli, and fungi [2]. 2-PE has been explored as a potential alternative to traditional chemical preservatives, particularly its application in fruit preservation [3]. In the pharmaceutical field, 2-PE serves as a precursor for the synthesis of various drugs, such as phenethyl acetate (a component of neurological medications) and certain cardiovascular agents [2]. In the food industry, 2-PE is widely used as a flavoring agent in beverages, confectionery, baked goods, and dairy products, imparting a rose-like aroma to these products [4]. In the personal care industry, 2-PE is a common fragrance ingredient in perfumes, cosmetics, soaps, and skin care products [4]. Given the broad range of applications described above, the development of efficient and cost-effective strategies for high-yield 2-PE production holds significant scientific value and commercial potential. In fermented beverages, 2-PE is also a key flavor compound in a variety of fermented products, including wine, beer, and bread. So, it is one of the key factors in determining the aromatic quality of brewed wine and fermented foods [5].

As a key aroma compound in brewed wine, 2-PE is highly valued for its rose-like odor [6]. However, its content in fermented beverages is generally low, typically below 0.1 g/L in distilled spirits (50% vol) [7,8]. Whisky, a typical distilled spirit, exhibits similarly low 2-PE content. The fermentation stage critically influences flavor development, as yeast produces a wide range of volatile compounds, including 2-PE, that shape the spirit’s sensory profile [7]. Consequently, enhancing 2-PE synthesis during whisky fermentation is key to improving its aromatic quality [9]. In whisky production, *S. cerevisiae* is selected as the preferred yeast strain due to its higher ethanol tolerance and better adaptability compared to other yeasts, such as *Candida*, *Kluyveromyces* [10,11,12,13]. Owing to these advantages, *S. cerevisiae* is regarded as a preferred microbial host strain for 2-PE production.

**Figure 1:**
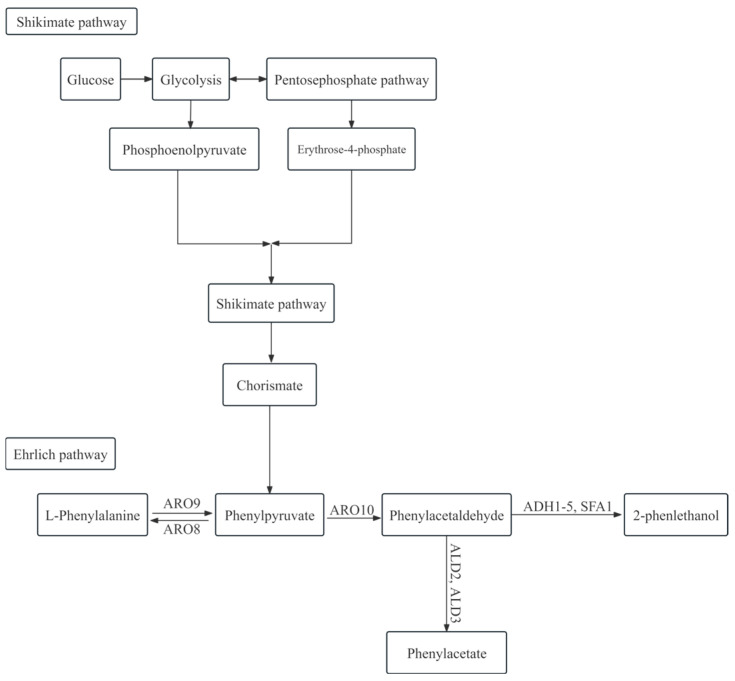
Shikimate pathway and Ehrlich pathway for 2-PE synthesis in *S. cerevisiae* [11,16].

2-PE is primarily synthesized via the shikimate pathway and the Ehrlich pathway in *S. cerevisiae* [14,15]. In the shikimate pathway, phosphoenolpyruvate (PEP) derived from glycolysis and erythrose 4-phosphate (E4P) derived from the pentose phosphate pathway are initially condensed to form 3-deoxy-D-arabino-heptulosonate-7-phosphate (DAHP). DAHP is then converted to phenylpyruvate through a series of enzymatic steps. Subsequently, phenylpyruvate is converted to 2-PE via decarboxylation and reduction [15,16], as shown in Figure 1. Although the shikimate pathway is widely present in microorganisms, it has low 2-PE yields with a lengthy metabolic route, numerous side branches, and multiple inhibitory effects [17]. In the Ehrlich pathway, L-phenylalanine (L-Phe) is transaminated to phenylpyruvate, then decarboxylated, obtaining phenylacetaldehyde, and finally reduced to 2-PE [4,18]. The research was focused on the Ehrlich pathway of biosynthesizing 2-PE due to its relatively high conversion efficiency and simple metabolic route [19,20].

In the Ehrlich pathway, the conversion of L-Phe to phenylpyruvate, catalyzed by Aro8 and Aro9, is the key step in the biosynthesis of 2-PE. Phenylpyruvate is then converted to phenylacetaldehyde by Aro10 [18,21,22], and phenylacetaldehyde is finally reduced to 2-PE by alcohol dehydrogenases (*ADH1*, *ADH2*, *ADH3*, *ADH4*, and *ADH5*) or formaldehyde dehydrogenase (*SFA1*) [23,24]. Liang et al. [25] demonstrated that overexpression of the *ARO10* gene in *S. cerevisiae* increased 2-PE yield by 16.3% compared to the wild-type strain. Kim et al. [20] constructed an engineered *S. cerevisiae* by deleting *ALD3* and overexpressing *ARO9* and *ARO10* to ferment 4.8 g/L 2-PE in a medium with 10 g/L L-Phe as the sole nitrogen source. A previous study reported that knockout of the *ARO8* gene altered the intracellular amino acid pool, consequently upregulating *ARO10* expression and enabling yeast to produce 2-PE in a glucose-based synthetic medium with ammonium sulfate as the sole nitrogen source [26]. Although most studies have focused on genetic improvement of *S. cerevisiae* for enhancing 2-PE synthesis, their application in distilled liquor has yet to be investigated.

The present study aimed to enhance 2-PE synthesis in *S. cerevisiae* through genetic improvement and to further increase 2-PE content in brewed wine by optimizing fermentation conditions. The SY-A8 strain with the ARO8 gene deletion was successfully constructed via CRISPR-Cas9 in this study. Subsequently, the fermentation process was optimized to increase 2-PE content in whisky. These findings provide a technical basis for improving the aromatic quality of whisky.

## 2. Results

### 2.1. Construction of Recombinant S. cerevisiae Strains

#### Construction of *ARO8* Gene Knockout Strain and PCR Validation

**Figure 2:**
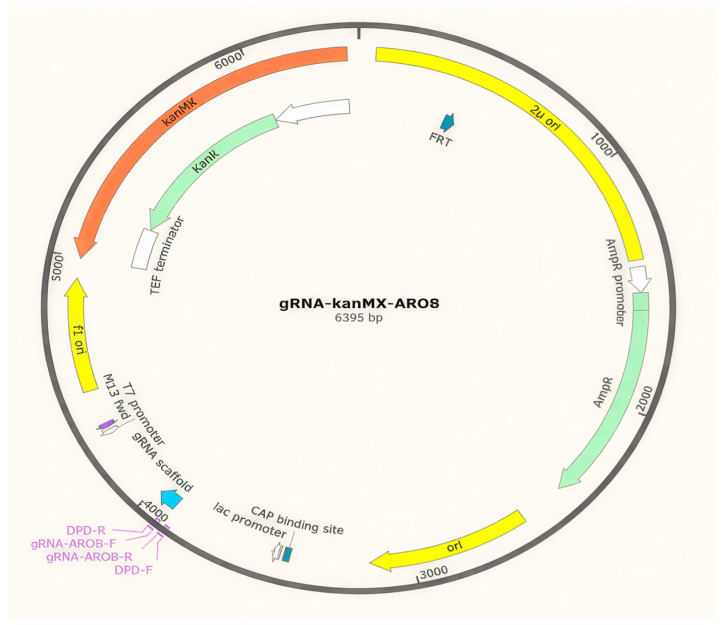
The recombinant plasmid p426-gRNA-ARO8-kanMX.

The recombinant plasmid p426-gRNA-ARO8-kanMX (6395 bp) contained the following key components: a pUC-derived origin of replication (ori) for propagation in E. coli; an ampicillin resistance gene (AmpR) for bacterial selection; a kanamycin resistance cassette (kanMX) for selection in *S. cerevisiae*; the *S. cerevisiae* SNR52 promoter driving expression of the single guide RNA (sgRNA); a 20-nucleotide guide sequence (5′-CATTGCAGACCATAGAAAGG-3′) targeting the *ARO8* gene; the sgRNA scaffold for Cas9 binding; and a terminator sequence for transcriptional termination. The schematic representation of the plasmid is shown in Figure 2.

**Figure 3:**
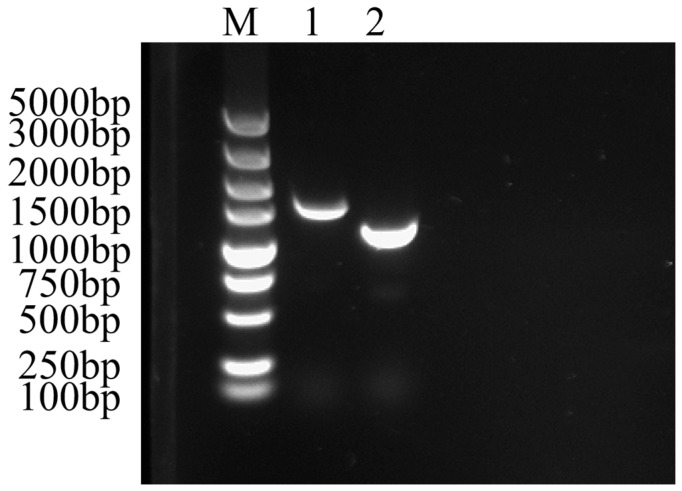
Construction results of recombinant *S. cerevisiae* strains. Lane M shows the 5000 bp marker. Verification results for SY-A8. Lane 1 is SY; Lane 2 is SY-A8.

Subsequently, electroporation was performed using the recombinant plasmid and donor DNA. Then, the recombinant strain was validated by PCR with primers ARO8-YZ-F and ARO8-YZ-R, as shown in Figure 3. The *ARO8* gene in SY-A8 was successful knockout of the 1130 bp (a 500 bp knockout) compared to 1630 bp (SY).

### 2.2. Effect of ARO8 Gene Knockout on 2-PE

**Figure 4:**
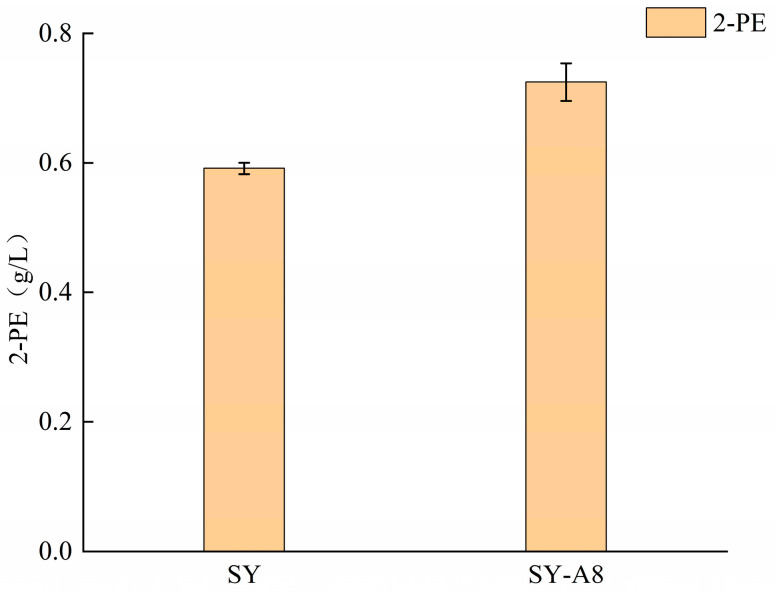
2-PE production by SY and SY-A8. Each experiment was conducted in triplicate. The *p*-value of less than 0.05 was considered statistically significant.

To evaluate the effect of *ARO8* knockout on 2-PE production, the content of 2-PE in whisky fermented by the SY and SY-A8 was measured by liquid chromatograph, and the results are shown in Figure 4. The alcohol contents and the corresponding 2-PE contents of whisky fermented by the original strain SY and the recombinant strain SY-A8 are presented in Tables S1 and S2, respectively. The 2-PE content produced by the SY-A8 was 0.73 g/L, an increase of 23.73% compared to the SY (0.59 g/L). The results indicate that the *ARO8* knockout strain effectively increased 2-PE content in whisky. Therefore, the recombinant strain SY-A8 can be used as the experimental strain for subsequent fermentation process optimization.

### 2.3. Relative Expression Levels of ARO8

**Figure 5:**
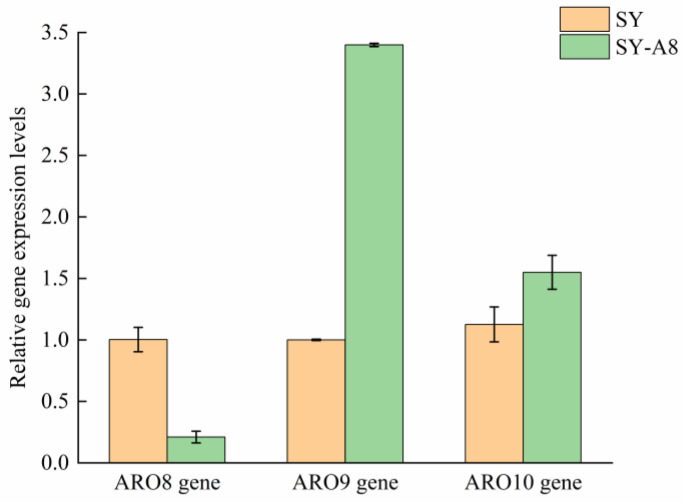
Relative expression levels of the *ARO8*, *ARO9* and *ARO10* genes in the SY and SY-A8 strains. Each experiment was conducted in triplicate. The *p*-value of less than 0.05 was considered statistically significant.

To confirm the transcriptional knockout of the *ARO8* gene, total RNA was isolated from the SY and SY-A8 strains, and cDNA was synthesized via reverse transcription. Subsequently, the relative expression levels of *ARO8* were determined by qPCR using the primers ARO8-F/ARO8-R, ARO9-F/ARO9-R, and ARO10-F/ARO10-R with the cDNA as a template. As shown in Figure 5, the *ARO8* gene in the SY-A8 strain was successfully knocked out, as evidenced by its markedly reduced expression level.

To further investigate whether the deletion of *ARO8* affects other key genes in the Ehrlich pathway, the expression levels of *ARO9* and *ARO10* in the SY-A8 strain were also examined. Both genes were found to be upregulated. Given that *ARO9* and *ARO10* encode key enzymes in the 2-PE biosynthetic pathway, their increased expression likely plays a crucial role in enhancing 2-PE yield.

### 2.4. Plasmid Loss Verification

**Figure 6:**
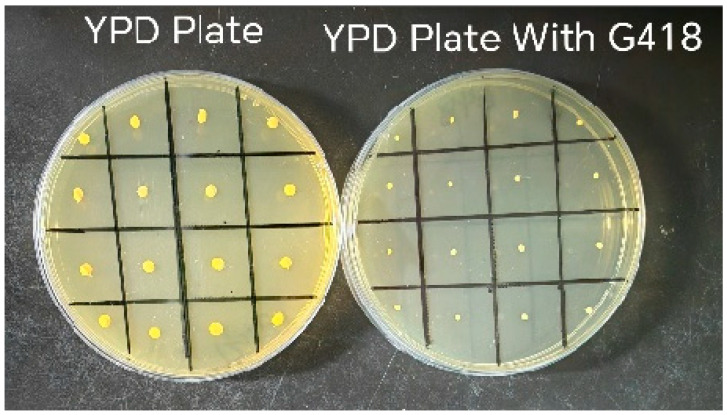
Verification of plasmid loss in recombinant strains.

The SY-A8 with the lost plasmid was screened by the photocopy plate method and is shown in Figure 6. The plasmid-depleted strain normally grew on the antibiotic-free plates, but slowly grew on the G418 sulfate plates. It indicates that the plasmid p426-gRNA.ARO8-kanMX has been successfully removed from the SY-A8.

### 2.5. Effect of ARO8 Gene Knockout on S. cerevisiae Growth

**Figure 7:**
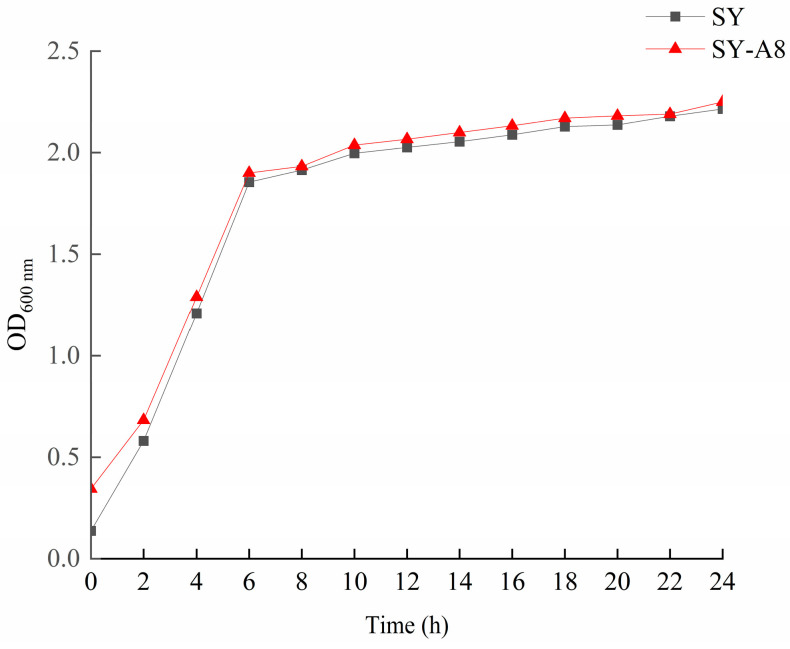
Growth curves of the original strain SY and recombinant strain SY-A8. Each experiment was conducted in triplicate. The *p*-value of less than 0.05 was considered statistically significant.

The growth curves of the original strain SY and recombinant strain SY-A8 are shown in Figure 7. The two strains exhibited similar growth rates, indicating that deletion of the *ARO8* gene does not affect *S. cerevisiae* growth.

### 2.6. Single-Factor Experiment Results

Through preliminary fermentation screening, the recombinant strain SY-A8, which exhibited the highest 2-PE yield, was selected for subsequent fermentation condition optimization. Single-factor experiments were performed to evaluate the effects of inoculum size, wort pH, sugar concentration, fermentation time, and L-Phe concentration on 2-PE production. The results are presented below.

#### 2.6.1. Effect of Different Inoculum Size on 2-PE in Whisky

**Figure 8:**
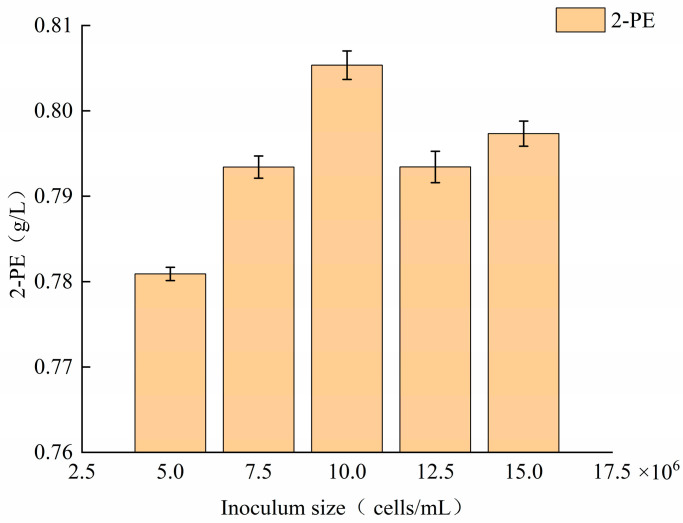
2-PE production by SY-A8 in different inoculum sizes. Each experiment was conducted in triplicate. The *p*-value of less than 0.05 was considered statistically significant.

The content of 2-PE in whisky was produced following the inoculum size, as shown in Figure 8. As there is an increase in the *S. cerevisiae* inoculum size from 5 × 10^6^ cells/mL to 15 × 10^6^ cells/mL, the yield of 2-PE in whisky continues to rise. At a lower *S. cerevisiae* inoculum size, the limited *S. cerevisiae* size resulted in slowing product accumulation and extending fermentation time. Although a higher *S. cerevisiae* inoculum size requires larger seed culture volumes and causes rapid nutrient depletion and massive by-product accumulation, it does not effectively improve the yield of 2-PE. The maximum content of 2-PE was approximately 0.81 g/L at an inoculum size of 10 × 10^6^ cells/mL.

#### 2.6.2. Effect of Wort pH on 2-PE in Whisky

**Figure 9:**
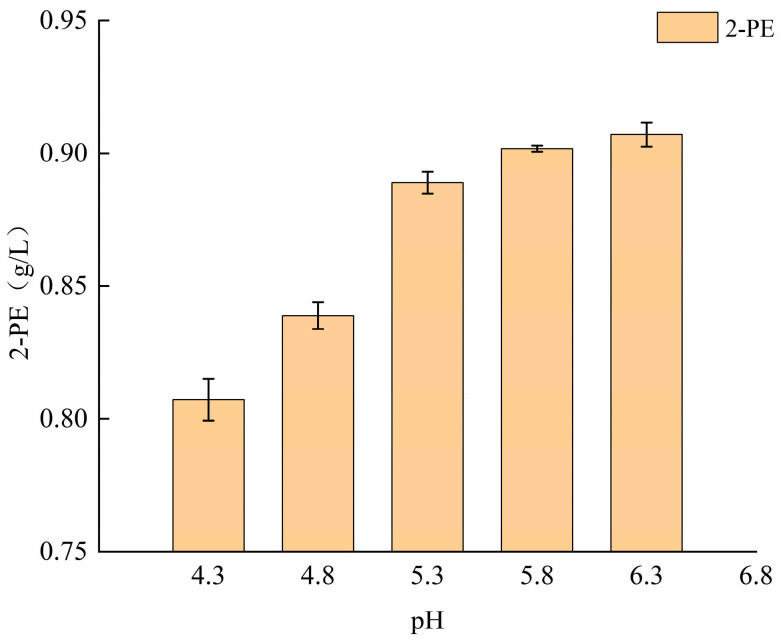
2-PE production by SY-A8 in different wort pH. Each experiment was conducted in triplicate. The *p*-value of less than 0.05 was considered statistically significant.

The content of 2-PE in whisky was produced under different wort pH levels, as shown in Figure 9. As the wort pH increases from 4.3 to 6.3, the yield of 2-PE in whisky continues to rise. At lower wort pH, the metabolic activity of *S. cerevisiae* was inhibited, which led to a decrease in the production of 2-PE. Conversely, at an optimal wort pH, the enzymes mediating the biosynthetic pathway of 2-PE display high catalytic activity, with *S. cerevisiae* exhibiting robust metabolic performance. The maximum 2-PE content was 0.91 g/L at optimal wort pH 6.3.

#### 2.6.3. Effect of Different Sugar Concentration on 2-PE in Whisky

**Figure 10:**
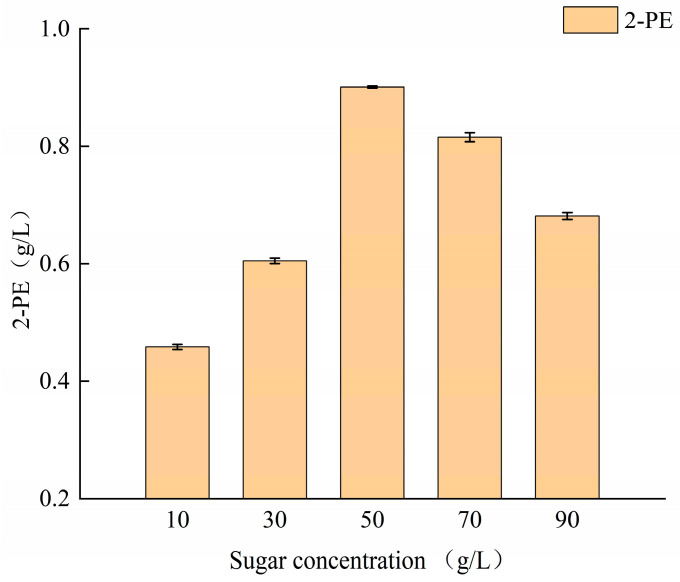
2-PE production by SY-A8 in different sugar concentrations. Each experiment was conducted in triplicate. The *p*-value of less than 0.05 was considered statistically significant.

The content of 2-PE in whisky was produced following the sugar concentration, as shown in Figure 10. As there is an increase in the sugar additions from 10 to 50 g/L, the yield of 2-PE in whisky continues to rise. This also improves the efficiency of converting sugar into 2-PE. When sugar additions exceed 50 g/L, the biosynthesis rate of ethanol was significantly accelerated, with sugars primarily utilized for ethanol production, which triggers a decrease in the production of 2-PE. The maximum content of 2-PE was 0.90 g/L at the optimal sugar concentration of 50 g/L.

#### 2.6.4. Effect of Fermentation Time on 2-PE in Whisky

**Figure 11:**
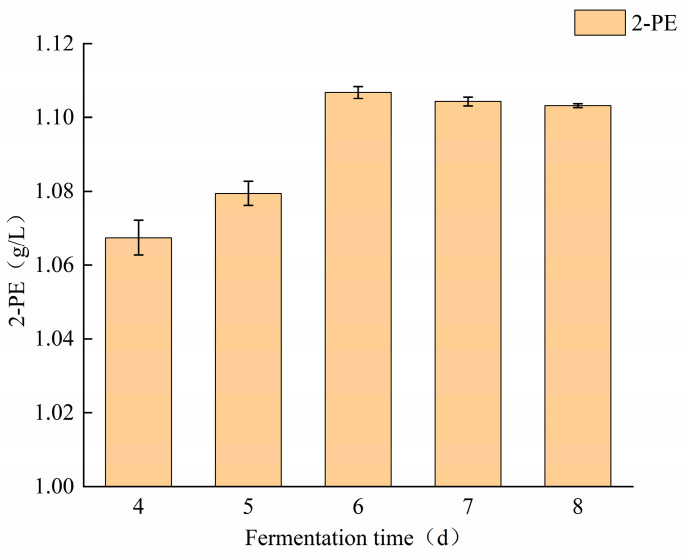
2-PE production by SY-A8 in different fermentation times. Each experiment was conducted in triplicate. The *p*-value of less than 0.05 was considered statistically significant.

The content of 2-PE in whisky produced under different fermentation times is shown in Figure 11. During the anaerobic fermentation from 4 to 6 days, under the condition of sufficient nutrients, the metabolic activity of yeast was maintained at a high level. So, the content of 2-PE was continually increased. After 6 days, the depletion of nutrients and accumulation of undesirable by-products led to decreased metabolic activity of the yeast. So, the content of 2-PE no longer increased significantly. The maximum content of 2-PE was 1.10 g/L at the optimal 6 days.

#### 2.6.5. Effect of L-Phe Concentration on 2-PE in Whisky

**Figure 12:**
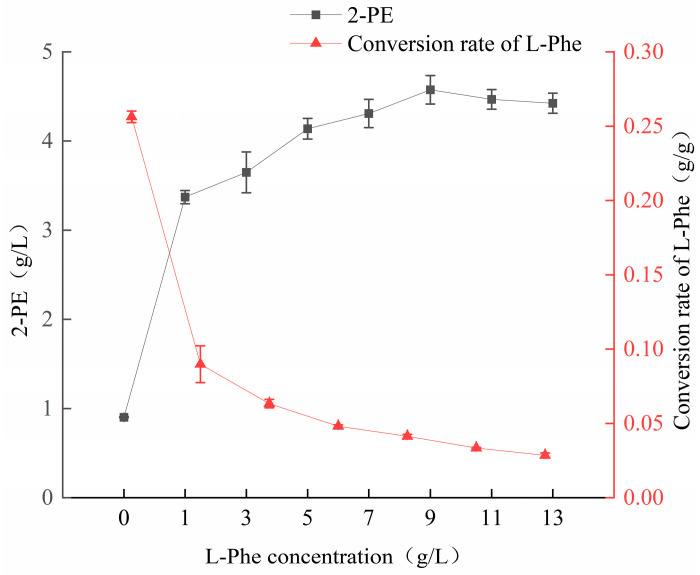
2-PE production by SY-A8 in different L-Phe concentrations. Each experiment was conducted in triplicate. The *p*-value of less than 0.05 was considered statistically significant.

The content of 2-PE in whisky produced under different L-Phe concentrations is shown in Figure 12. As the concentration of L-Phe increased from 0 to 13 g/L, the content of 2-PE continuously increased. Although adding L-Phe enhances 2-PE production, the conversion rate of 2-PE from L-Phe continually decreased. So, the maximum molar yield of 2-PE from L-Phe was 0.25 g/g (or 0.34 mol/mol) at the L-Phe concentration of 1 g/L, and the content of 2-PE was 3.31 g/L, an increase of 267.78% compared with the 0 g/L L-Phe. These experimental data indicate that the addition of L-Phe could effectively enhance the biosynthetic pathways of 2-PE. Therefore, considering both 2-PE content and the conversion rate of 2-PE from L-Phe, the optimal concentration of L-Phe was 1 g/L.

### 2.7. Optimization of Fermentation Conditions Using Response Surface Methodology

#### 2.7.1. Response Surface Methodology Model and Statistical Significance Analysis

The Box–Behnken design was employed for the response surface methodology, with investigating factors including sugar concentration (A), fermentation time (B), and L-Phe concentration (C). The response value was the 2-PE content (Y) in the whisky. The experimental results are detailed in Table 1.

The experimental data were analyzed using Design Expert 13 to obtain a multiple quadratic regression equation:

Y = 3.5335 − 0.1169A + 0.0394B − 0.0095C + 0.1508AB + 0.1254AC + 0.0007BC − 0.2628A^2^ − 0.2503B^2^ + 0.2694C^2^.

#### 2.7.2. Variance and Confidence Analysis of 2-PE Content in Whisky

A further analysis of variance was conducted on the results of each experimental group from Table 1, and the findings are presented in Table 2.

As shown in Table 2, a quadratic model was applied to regress the 2-PE content in whisky. The model showed a highly significant difference with a *p*-value < 0.01, indicating a robust fit and suitability for subsequent optimization analyses. The lack-of-fit test had a *p*-value > 0.05, suggesting that the quadratic model provided an adequate fit across the regression range, effectively modeling the 2-PE content in whisky. An analysis of F-values revealed that the most influential factor was sugar concentration (A), fermentation time (B) and L-Phe concentration (C). In the significance analysis, using a threshold of *p* < 0.05, it was found that the linear terms A, B, quadratic terms A^2^, B^2^ and C^2^, and the interaction terms AB and AC had significant effects on the 2-PE content in whisky (*p* < 0.05). The model’s R^2^ value was 0.9918, indicating a strong correlation between the actual and predicted values, with 99.18% of the variance in the 2-PE content explained by the model. The adjusted R^2^ value was 0.9813, suggesting that 98.13% of the variability was accounted for by the investigated variables, demonstrating that the optimized model effectively captures the process conditions. The predicted R^2^ value was 0.8961, with the difference between the predicted and actual R^2^ values being less than 0.2, further validating the model’s accuracy and low error rate [27].

#### 2.7.3. Response Surface Analysis and Verification Test of 2-PE Content in Whisky

The analysis results indicate that the *p*-values for the sugar concentration (A) and fermentation time (B) in the first-order term, A^2^, B^2^ and C^2^ in the second-order term, AB and AC in the interaction term are all below 0.05, demonstrating their significant influence on 2-PE content. Generally, a lower coefficient of variation (CV) corresponds to higher experimental reliability and precision. The CV% of this regression model is 1.06, indicating high reliability and accuracy in the response surface experiment; the adequate precision is 38.95, a value greater than four is considered reasonable. These findings confirm that the model exhibits high reliability, accuracy, and good adaptability [28].

**Figure 13:**
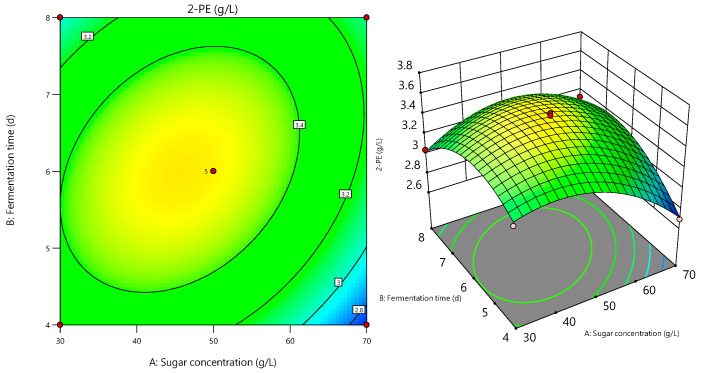
Effect of sugar concentration and fermentation time on the content of 2-PE.

**Figure 14:**
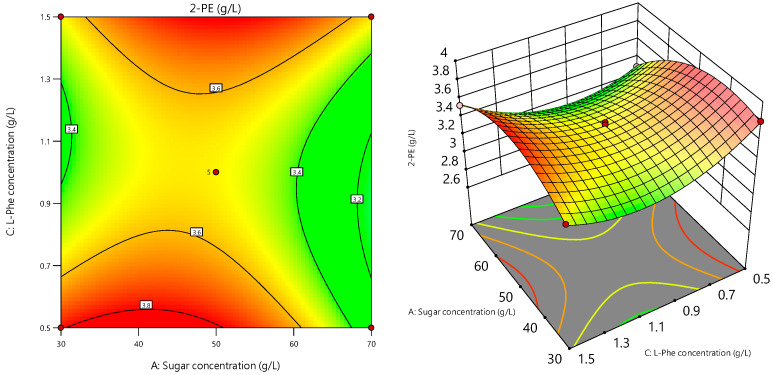
Effect of sugar concentration and L-Phe concentration on the content of 2-PE.

**Figure 15:**
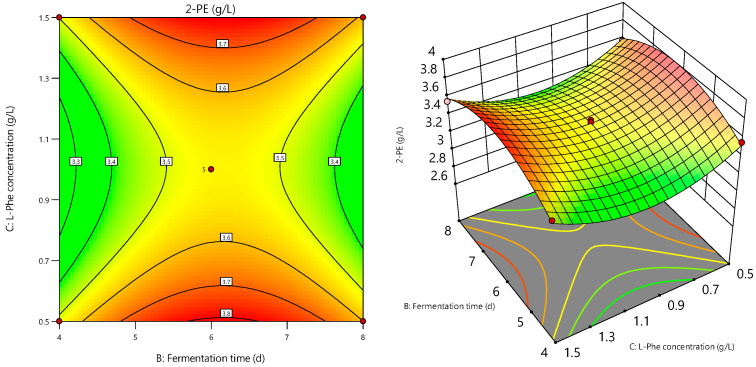
Effect of fermentation time and L-Phe concentration on the content of 2-PE.

Based on the results in Table 2, three-dimensional response surface plots were generated using Design Expert to visualize the interaction effects between the investigating factors AB, AC, and BC, as shown in Figure 13, Figure 14 and Figure 15, respectively. According to the fundamental principles of the response surface methodology, circular contour lines typically indicate a minimal interaction between two factors, while elliptical or saddle-shaped contour lines suggest a significant interaction. Furthermore, a steep response surface indicates strong interaction between factors, whereas a flatter surface suggests weaker interaction [29,30].

As shown in Figure 13, at a constant fermentation time, 2-PE content first increased and then decreased as sugar concentration increased, and exhibited a similar trend at a constant sugar concentration as fermentation time was extended. The optimal sugar concentration supplied sufficient carbon sources for yeast growth, while excessive sugar induced osmotic stress and reduced metabolic activity. Short fermentation time led to insufficient 2-PE synthesis, whereas prolonged fermentation caused nutrient depletion and by-product accumulation. There was a significant interaction between sugar concentration and fermentation time, as reflected by elliptical contours and pronounced surface curvature. These two factors jointly regulated 2-PE biosynthesis, and their coordinated optimization enhanced 2-PE production.

As shown in Figure 14, at a constant L-Phe concentration, 2-PE content increased gradually as sugar concentration increased. At a constant sugar concentration, the 2-PE yield first increased and then decreased with increasing L-Phe concentration. The optimal L-Phe concentration served as a key precursor to facilitate 2-PE synthesis, while excessive addition inhibited yeast metabolism. The optimal sugar concentration strengthened microbial metabolism and further promoted product accumulation. Curved contours and a steep response surface revealed a significant interaction between sugar concentration and L-Phe concentration. When properly balanced, these two substrate concentrations effectively enhanced 2-PE production.

As shown in Figure 15, at a constant L-Phe concentration, 2-PE content first increased and then decreased as fermentation time prolonged. When fermentation time was constant, 2-PE content also rose first and fell later with the increase of L-Phe concentration. Yeast sufficiently converted the precursor within an appropriate fermentation time. It failed to achieve complete synthesis under insufficient time, whereas prolonged fermentation led to nutrient depletion and by-product accumulation. As a key precursor for 2-PE synthesis, a suitable L-Phe concentration can significantly promote product formation, while excessive concentration will cause metabolic inhibition. The two factors have a significant interaction, as evidenced by elliptical contours and obvious response surface changes. It is necessary to synergistically optimize fermentation time and L-Phe addition to achieve efficient accumulation of 2-PE.

Based on the above analysis, the optimal process conditions determined by the response surface model were as follows: sugar concentration of 46.28 g/L, fermentation time of 6.26 days, and L-Phe concentration of 1.43 g/L. Under these conditions, the maximum 2-PE content predicted by Design Expert 13 was 3.71 g/L. To validate the reliability of the predictions obtained by the response surface methodology, scale-up fermentation experiments were conducted under the above-mentioned conditions at 1 L scales. Specifically, with a sugar concentration of 46.30 g/L, a fermentation time of 6 days, and an L-Phe concentration of 1.43 g/L, the contents of 2-PE were 3.68 g/L. The experimental validation results was close to the predicted values, indicating that the response surface model established by the above experimental method is highly reliable and can be applied to the actual fermentation production of whisky. However, further pilot-scale or industrial-scale fermentation studies are needed to confirm the applicability of these optimized conditions in large-scale whisky production. 

### 2.8. Sensory Evaluation and Off-Flavor Analysis of 2-PE Aroma Whisky

**Figure 16:**
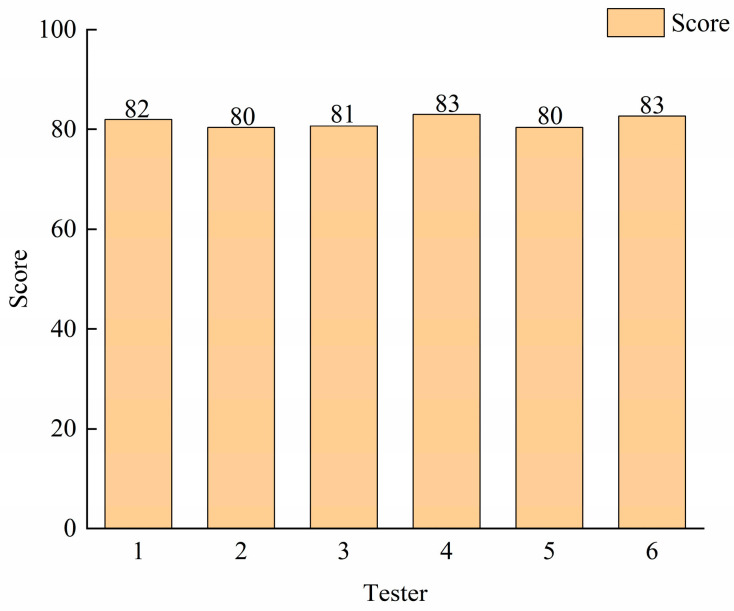
Sensory and off-flavor evaluation of whisky.

To evaluate the sensory characteristics and consumer acceptance of whisky produced by the recombinant strain SY-A8, the sensory properties (including taste and flavor) of the samples fermented under the optimal fermentation conditions determined by response surface methodology were systematically assessed in this study. The results are presented in Figure 16.

As shown in Figure 16, the sensory scores provided by six trained panelists were consistent, ranging from 80 to 83, with an average score of approximately 81.5. The evaluation results indicated that the whisky fermented by the recombinant strain SY-A8 exhibited a pronounced rose-like aroma and a rich alcoholic note that were well coordinated with the malt character; however, a slight bitterness was perceived in taste. We speculate that other higher alcohols produced by *S. cerevisiae* during fermentation may contribute to this slight bitterness in the spirit. Overall, the panelists gave relatively high ratings for the whisky’s taste, suggesting that the whisky produced by the recombinant strain is palatable and acceptable to consumers. The consistency of the panelists’ scores further highlights the stability and reliability of the final product quality, thus making it suitable for the development of 2-PE rose-aroma beverages.

## 3. Discussion

The primary biosynthetic pathways of 2-PE in *S. cerevisiae* SY-A8 are the shikimate pathway and the Ehrlich pathway [31]. The Ehrlich pathway, with its short metabolic route, is regulated by transaminases, decarboxylases, and alcohol dehydrogenases [32,33], as shown in Figure 1. The transaminase activity in this pathway is closely linked to cellular physiological growth, as it is tightly integrated with the TCA cycle through transamination with α-ketoglutarate [34]. The phenylpyruvate decarboxylase encoded by *ARO10* primarily catalyzes the conversion of phenylpyruvate to phenylacetaldehyde [34,35]. A previous study demonstrated that in ∆ARO8 and ∆ARO9 strains, the expression of *ARO10* increased significantly by up to sixfold, and the 2-PE production in ∆*ARO8* and ∆*ARO9* increased by 85% and 38%, respectively [36]. To investigate whether *ARO8* deletion affects the expression of other genes in the Ehrlich pathway, we measured the expression levels of *ARO9* and *ARO10* by qPCR. The results showed that *ARO9* and *ARO10* were approximately upregulated by 3.4-fold and 1.4-fold, respectively, in the SY-A8 strain compared to the original strain SY. We suggest that deletion of *ARO8* reduces the conversion of phenylpyruvate to L-Phe, while the increased expression of *ARO10* promotes the formation of phenylacetaldehyde, thereby enhancing 2-PE synthesis via carbohydrate metabolism. This interpretation suggests that the observed changes in gene expression contribute to increased 2-PE production, though further research is warranted to confirm this mechanistic relationship.

However, the Ehrlich pathway relies on exogenous L-Phe supplementation, which is economically unfavorable for industrial-scale production. In contrast, the shikimate pathway enables de novo synthesis of 2-PE directly from glucose, bypassing the need for expensive precursor addition [34,37]. Recent studies have demonstrated that the two pathways can operate cooperatively under specific conditions. Zhang et al. [38] reported that corn steep liquor activates the expression of both shikimate and Ehrlich pathway genes (including aro4, aro8, and aro9), thereby promoting 2-PE synthesis through both routes. Yang et al. [34] demonstrated that when the shikimate and Ehrlich pathways coexist, the theoretical conversion yield of 2-PE can be exceeded, achieving a titer of 12.1 g/L in fed-batch fermentation. Beyond transcriptional regulation, additional layers of control, such as post-translational modifications of key enzymes (e.g., aro8, aro9, and aro10) and allosteric feedback inhibition by pathway intermediates, may also play important roles in modulating 2-PE biosynthesis. Furthermore, transcription factors including Aro80, Cat8, and Mig1 are known to regulate the expression of *ARO9* and *ARO10* in response to nitrogen availability and carbon source, suggesting that the regulatory network governing the Ehrlich pathway is multifaceted and warrants deeper investigation [14,21,22,39]. In our study, *ARO8* deletion combined with process optimization yielded 3.68 g/L 2-PE, a substantial improvement over the original strain. Further enhancement could be achieved by co-engineering the shikimate pathway, for example, by relieving feedback inhibition of *ARO3* and *ARO4* and strengthening precursor supply [35,39].

In previous studies, Wu et al. [40] constructed a high 2-PE-yield strain via mutation breeding. After optimizing aerobic and anaerobic fermentation processes, the maximum content of 2-PE was 1.69 g/L in distilled liquor (30% vol). Our results showed that the 2-PE content in whisky fermented by SY-A8 was 0.73 g/L, an increase of 23.73% compared to SY. This indicated that the upregulated expression of *ARO10* promoted 2-PE biosynthesis. The fermentation conditions of SY-A8 were optimized through single-factor experiments and the Box–Behnken design. The optimal conditions were a sugar concentration of 46.30 g/L, a fermentation time of 6 days, and an L-Phe concentration of 1.43 g/L. The high 2-PE aroma whisky was brewed with a higher 2-PE content of 3.68 g/L in a 1 L fermenter at the optimal conditions. In conclusion, *ARO8* deletion combined with fermentation optimization enabled *S. cerevisiae* to efficiently produce 2-PE, increasing the concentration of 2-PE in whisky to 3.68 g/L.

Our results indicated that deletion of the *ARO8* gene contributed to the upregulation of *ARO9* and *ARO10*, and improved the production of 2-PE in distilled liquor. Although these findings support the effectiveness of the strain modification, a more systematic investigation of the related metabolic flux and comprehensive molecular regulatory mechanism will be further explored and supplemented in future studies to achieve a deeper understanding of 2-PE biosynthesis.

## 4. Materials and Methods

### 4.1. Materials and Reagents

#### 4.1.1. The Construction of Strains and Plasmids

The strains and plasmids used in this study are listed in Table S3. *Escherichia coli* match-T1 with the p426-gRNA-ARO8-kanMX plasmid was grown in LB medium supplemented with 100 µg/mL ampicillin (Aladdin, Shanghai, China) at 37 °C for 12–16 h. *S. cerevisiae* SY was incubated at 30 °C for 12 h in YPD medium containing 100 µg/mL G418 sulfate (Aladdin, Shanghai, China) to select for positive transformants [41].

#### 4.1.2. Primer Design

The primers used in this study are listed in Table S4. The ARO8 gene sequence of S. cerevisiae (S288c) was obtained from the National Center for Biotechnology Information (NCBI). The primers were designed using SnapGene 5.2.0 software and synthesized by Shanghai Bioengineering Technology Co., Ltd. (Shanghai, China) [29].

### 4.2. Construction of the CRISPR-Cas9 System

CRISPR-Cas9 is a gene editing technology involving two fundamental components: a guide RNA matching the target gene, and a nucleic acid endonuclease Cas9, causing a double-stranded DNA break and modifying the genome [42].

When constructing the Cas9 expression plasmid, the fragments containing the Cas9 gene and the bleomycin resistance gene (BleoR gene) were obtained by PCR amplification of the p414-Cas9 plasmid and pPICZ (alpha) plasmid as templates [43]. The purified fragments were ligated in vitro with a homologous recombination kit. These ligation products were then transformed into competent *E. coli* cells to screen for positive colonies. Subsequently, the Cas9 expression plasmid p414-Cas9-BleoR was purified using a plasmid extraction kit (Thermo Fisher, Shanghai, China) [29].

To construct the gRNA expression plasmid, the original target site was substituted with the *ARO8* specific sequence. SnapGene 5.2.0 software was used to identify the protospacer adjacent motif (PAM; 5′-NGG-3′ or 5′-NAG-3′) within the *ARO8* gene, and a 20-nucleotide guide sequence was selected immediately upstream of the PAM. The 60 bp fragment containing the 20 nt *ARO8* target sequence was obtained by overlap extension PCR (98 °C for 3 min, followed by 30 cycles consisting of 98 °C for 10 s, 55 °C for 5 s, 72 °C for 10 s, and a final extension at 72 °C for 2 min). The vector fragment (6375 bp) was obtained by PCR amplification with primers DPD-F/DPD-R (98 °C for 3 min, followed by 30 cycles consisting of 98 °C for 10 s, 55 °C for 15 s, 72 °C for 7 min, and a final extension at 72 °C for 9 min) and template gRNA plasmid p426-gRNA-kanMX [31]. A pair of primers, each containing a 20 bp sequence homologous to the target regions, was designed for the 6395 bp vector fragment and the 60 bp fragment. Following purification, in vitro assembly of these two fragments was carried out using the ClonExpress^®^ II One Step Cloning Kit (Vazyme, Nanjing, China). The ligation product was then introduced into Escherichia coli match-T1 competent cells. Through this process, we obtained the gRNA expression plasmid p426-gRNA-ARO8-kanMX, which is designed to specifically recognize the *ARO8* target sequence [29].

**Figure 17:**
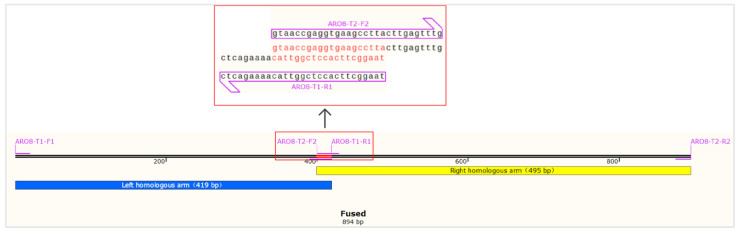
Schematic diagram of donor DNA construction.

*ARO8* Donor DNA Design (Figure 17): Using the SY genome as a template, the upstream and downstream homologous arms of the *ARO8* target sequence were amplified by PCR with primer pairs ARO8-T1-F1/R1 and ARO8-T2-F2/R2 (98 °C for 3 min, followed by 30 cycles consisting of 98 °C for 10 s, 55 °C 15 s, 72 °C for 10 s, and a final extension at 72 °C for 1 min), respectively (20 bp of homology was set at the 5′end of primers ARO8-T1-R1 and ARO8-T2-F2). Subsequently, the two fragments were connected by overlap extension PCR (98 °C for 3 min, followed by 30 cycles consisting of 98 °C for 10 s, 55 °C for 15 s, 72 °C for 1 min, and a final extension at 72 °C for 3 min). Finally, the *ARO8* donor DNA was obtained [29].

### 4.3. Electro Transformation of SY

The purified p414-Cas9-BleoR plasmid was transformed into the SY by electrotransformation. The transformed *S. cerevisiae* were cultured in YPD medium (200 µg/mL Zeocin™) (Thermo Fisher, Shanghai, China) at 30 °C for 48 h. Then, the positive transformants were selected by colony PCR. The SY with the p414-Cas9-BleoR plasmid was made into an electrocompetent yeast. It was mixed with p426-gRNA-ARO8-kanMX plasmid and donor DNA at a 1:10 (*v*/*v*) ratio to perform electroporation (Electroporation apparatus, 1.5 kV, 5 ms, Thermo Fisher, Shanghai, China). The transformed *S. cerevisiae was* cultured in YPD medium (100 µg/mL G418 sulfate) at 30 °C for 1–2 days. Then, the positive transformants were selected by colony PCR [29]. (Zeocin™: Thermo Fisher, Shanghai, China).

### 4.4. Screening and Validation of the Knockout Strain

The positive transformants were validated by PCR with primers ARO8-YZ-F and ARO8-YZ-R (3 min at 98 °C, followed by 30 cycles consisting of 98 °C for 10 s, 55 °C for 15 s, 72 °C for 2 min, and a final extension at 72 °C for 4 min) [29].

### 4.5. Fermentation and Determination of 2-PE

#### 4.5.1. Wort Preparation

Malt powder was mixed with water at a ratio of 1:4 (kg/L) and stirred to initiate saccharification. The saccharification process consisted of heating the mixture sequentially at 53 °C for 60 min, 65 °C for 60 min, and finally at 72 °C for 20 min. The resulting mixture was then filtered through four layers of sterile gauze. After preparation, the wort medium was stored at −20 °C until further use [29].

#### 4.5.2. Yeast Inoculation and Fermentation

The wort was prepared and adjusted to a concentration of 8% (*w*/*v*), and sugar was added to 50 g/L. The broth was brought to a final volume of 100 mL in a sterile 100 mL graduated cylinder. The inoculum suspension was centrifuged at 10,000 rpm for 5 min, and the cell pellet was collected. The pellet was washed twice with sterile deionized water and then resuspended in the fermented wort. Both strains, SY and SY-A8, were inoculated into the wort broth at an inoculum size of 5 × 10^6^ cells/mL. The cultures were then incubated at 28 °C for 7 days. The fermentation broth was gently shaken every 24 h throughout the incubation period to maintain homogeneous fermentation. After fermentation, the 2-PE content was measured by liquid chromatography.

#### 4.5.3. Determination Methods of 2-PE

The content of 2-PE in whisky was determined using an external standard method by high-performance liquid chromatography (HPLC). The chromatographic conditions were as follows: chromatographic column: Ultimate XB-C18 (4.6 mm × 250 mm, 5 μm); column temperature: 25 °C; mobile phase: methanol–water (55:45, *v*/*v*); flow rate: 1.0 mL/min; detection wavelength: 210 nm. Prior to analysis, the samples were diluted with the mobile phase and filtered through a 0.45 μm membrane [44].

### 4.6. Quantification of the Relative Expression Levels of the ARO8 Gene

Total RNA was extracted from the yeast strains using a total RNA extraction kit (Sangon, Shanghai, China). The relative expression level of the *ARO8* gene was then determined by qPCR using total RNA extracted from SY and SY-A8 as templates. The qPCR (30 s at 95 °C, and then 40 cycles of 95 °C for 10 s, 55 °C for 15 s, 72 °C for 15 s) with template of total RNA extracted from the SY and SY-A8 [29].

### 4.7. Discard Plasmids

The gene knockout strains were cultured in 5 mL of YPD broth at 30 °C for 24 h. They were then subcultured for more than 15 generations. Subsequently, the plasmid-depleted strain was screened on YPD medium containing 100 µg/mL G418 sulfate [29].

### 4.8. Determination of Growth Curve

The yeast seed culture was inoculated into 100 mL YPD medium and incubated at 30 °C, 200 r/min. Then, OD_600nm_ was determined every 2 h [43].

### 4.9. Optimization of Fermentation Conditions [29]

#### 4.9.1. Fermentation with Different *S. cerevisiae* Inoculum Size

The SY-A8 was inoculated in 8% (*w*/*v*) wort, adding 50 g/L sugar with inoculum size 5 × 10^6^, 7.5 × 10^6^, 10 × 10^6^, 12.5 × 10^6^ and 15 × 10^6^ cells/mL, respectively. Then, it was fermented at 28 °C for 7 days.

#### 4.9.2. Fermentation with Different Wort pH

The pH of 8% (*w*/*v*) wort broth, adding 50 g/L sugar, was adjusted to 4.3, 4.8, 5.3, 5.8 and 6.3, respectively. Then, it was fermented by SY-A8 at 28 °C for 7 days.

#### 4.9.3. Fermentation with Different Sugar Concentration

The 8% (*w*/*v*) wort broth was added with different sugar concentrations of 10, 30, 50, 70, and 90 g/L, respectively. Then, it was fermented by SY-A8 at 28 °C for 7 days.

#### 4.9.4. Fermentation with Different Fermentation Times

The 8% (*w*/*v*) wort, adding 50 g/L sugar, was fermented by SY-A8 at 28 °C for 4, 5, 6, 7, and 8 days, respectively.

#### 4.9.5. Fermentation with Different L-Phe Concentration

The 8% (*w*/*v*) wort, adding 50 g/L sugar, was added with different L-Phe concentrations of 0, 1, 3, 5, 7, 9, 11, and 13 g/L, respectively. Then, it was fermented by SY-A8 at 28 °C for 6 days.

### 4.10. Response Surface Optimization Experiment

A total of 17 experiments were conducted by the Box–Behnken design for the response surface methodology. As shown in Table 3, the design included three factors at three levels: sugar concentration (A), fermentation time (B), and L-Phe concentration (C). The levels were set at −1, 0, and 1, with the 2-PE content (Y) as the response variable. This approach was employed to optimize the experimental conditions through response surface optimization.

### 4.11. Sensory Evaluation and Off-Flavor Analysis of Whisky

The organoleptic characteristics of samples fermented under the optimal conditions determined by response surface methodology were evaluated by a trained sensory panel consisting of six members with expertise in brewing science: two supervisors, two corporate wine tasters, and two graduate students. The sessions were conducted in a well-lit, quiet, and controlled laboratory environment to minimize external interference. Following established sensory protocols for fermented beverages, the samples were assessed across four primary dimensions, including clarity, color, aroma, and flavor, based on the scoring criteria specified in Table 4. The final sensory score for each attribute was calculated as the arithmetic mean of all individual ratings, rounded to the nearest integer [45,46,47].

### 4.12. Data Processing

Due to the varying degrees of fermentation in the experimental samples, the results were uniformly converted and analyzed under a 50% vol alcohol system [29]. Each experiment was conducted in triplicate. Significance analysis was performed using SPSS v.27.0; a *p*-value of less than 0.05 was considered statistically significant. Origin v.10.1 and other software were used for plotting bar charts and line charts. 

## 5. Conclusions

The SY-A8 with gene knockout of *ARO8* was successfully constructed by CRISPR-Cas9 in this study. Knockout of the *ARO8* gene not only reduced the expression of *ARO8* but also simultaneously upregulated the expression of *ARO9* and *ARO10* in SY-A8. Then, the fermentation conditions of SY-A8 were optimized through single-factor experiments and the Box–Behnken design. The optimal conditions were a sugar concentration of 46.30 g/L, a fermentation time of 6 days, and an L-Phe concentration of 1.43 g/L. The high 2-PE aroma whisky was brewed with a higher 2-PE content of 3.68 g/L in a 1 L fermenter at the optimal conditions, an increase of 404.11% compared to the pre-optimization level. These findings suggest that genetic modification of the Ehrlich pathway in *S. cerevisiae* is an effective strategy for boosting 2-PE biosynthesis. This study provides a technical basis for improving the aroma of whisky and other brewed wines. Future research will focus on exploring combinatorial genetic modifications and scaled-up fermentation processes to promote industrial application.

## Figures and Tables

**Table 1:** Response surface test design and results.

		Factors		
Run	Sugar Concentration (g/L)	Fermentation Time (d)	L-Phe Concentration (g/L)	2-PE (g/L)
1	50	8	1.5	3.5533
2	50	4	1.5	3.5292
3	70	6	0.5	3.2960
4	30	4	1	3.2113
5	50	4	0.5	3.5533
6	50	6	1	3.5425
7	50	6	1	3.5079
8	50	6	1	3.5309
9	50	6	1	3.5191
10	30	8	1	3.0446
11	70	6	1.5	3.5316
12	70	4	1	2.6946
13	50	8	0.5	3.5745
14	70	8	1	3.1312
15	30	6	1.5	3.5334
16	50	6	1	3.5671
17	30	6	0.5	3.7995

**Table 2:** Response surface regression model analysis of variance.

	Sum of Squares	df	Mean Square	F-Value	*p*-Value	Significance
Model	1.11	9	0.1231	94.38	<0.0001	**
A	0.1094	1	0.1094	83.87	<0.0001	**
B	0.0124	1	0.0124	9.53	0.0177	*
C	0.0007	1	0.0007	0.5507	0.4822	
AB	0.0910	1	0.0910	69.79	<0.0001	**
AC	0.0629	1	0.0629	48.27	0.0002	**
BC	2.117 × 10^−6^	1	2.117 × 10^−6^	0.0016	0.9690	
A^2^	0.2907	1	0.2907	222.95	<0.0001	**
B^2^	0.2638	1	0.2638	202.33	<0.0001	**
C^2^	0.3056	1	0.3056	234.41	<0.0001	**
Residual	0.0091	7	0.0013			
Lack of Fit	0.0070	3	0.0023	4.52	0.0896	
Pure Error	0.0021	4	0.0005			
Cor Total	1.12	16				

Note. **: highly significant (*p* < 0.01); *: significant; significant (*p* < 0.05).

**Table 3:** Coded and actual values of factors in Box–Behnken design.

		Levels	
Factors	−1	0	1
Sugar concentration (g/L)	30	50	70
Fermentation time (d)	4	6	8
L-Phe concentration (g/L)	0.5	1	1.5

**Table 4:** Sensory evaluation criteria for the fermented beverage.

Attribute	Scoring Criteria	Score (Points)
Clarity and Color (20 points)	Pure color, clear and bright	15~20
	Pure color and clear	10~14
	Uniform color, clear and transparent	<10
Aroma (40 points)	Pronounced rose-like aroma, rich alcoholic note, well-coordinated with malt aroma	33~40
	Rose-like aroma and alcoholic note, coordinated with malt aroma	24~32
	Weak rose-like aroma and alcoholic note, poorly coordinated with malt aroma	<24
Flavor (40 points)	Balanced and smooth flavor, mellow taste, no off-flavors	33~40
	Balanced flavor, mellow taste, slight bitterness	24~32
	Unbalanced flavor, bitterness or off-flavors	<24

## Data Availability

The original contributions presented in the study are included in the article. Further inquiries can be directed to the corresponding author.

## Supplementary Materials

The following supporting information can be downloaded at: https://www.mdpi.com/article/10.3390/ijms27114759/s1.

## Abbreviations

The following abbreviations are used in this manuscript:

2-PE	2-phenylethanol
L-Phe	L-phenylalanine
