# Supplementary material for: Fermentation Process Optimization for High 2-Phenylethanol Aroma Whisky
Source: Int J Mol Sci. 2026 May 25;27(11):4759. doi: 10.3390/ijms27114759 (PMC13256722; doi:10.3390/ijms27114759)
Supplement: Supplementary file 1 [file ijms-27-04759-s001.zip › ijms-4253180-supplementary.pdf]

Table S1 Ethanol content in original strains and recombinant strains.

strains	Ethanol (% Vol)
SY	5.4
SY-A8	5.25

Table S2-PE production by the original and recombinant strains.

Strains	2-PE(g/L)
SY	0.59123±0.00903
SY-A8	0.7246±0.02882

Table S3 Strains and plasmids used in this study

Strains or plasmids	Relevant characteristics	Reference or provider
Strains		
SY	diploid yeast strain	This lab
SY-A8	Δ*ARO8-/-,*diploid yeast strain	This study
Plasmids		
p414	TEF1p-Cas9-CYC1t	This lab
pPICZ (alpha)	AOX1-BleoR	This lab
p414-Cas9-BleoR	TEF1p-Cas9-BleoR-CYC1t	This lab[1]
pUG6	kanMX	This lab
P426	SNR52p-gRNA-CAN1.Y-SUP4t	This lab[1]
p426-gRNA-*ARO8*-kanMX	SNR52p-gRNA-ARO8-Y-SUP4t- kanMX	This study

References:

1. Wang, Z.; He, J.; Lang, S.; Zhou, S. Construction of *LEU1* gene deleted *Saccharomyces cerevisiae* based on CRISPR-Cas9 system for brewing low degree of drunkenness rice wine. *China Brewing* **2024**, 43, 62-67, doi:10.11882/j.issn.0254-5071.2024.04.009.

Table S4 List of the Primer sequences used in this study

Primers	sequence (5′→ 3′) [a]
For disruption cassette construction	
ARO8-gRNA-F	CCTTTCTATGGTCTGCAATGGTTTTAGAGCTAGAAATAGC

ARO8-gRNA-F	CATTGCAGACCATAGAAAGGGATCATTTATCTTTCACTGC
DPD-F	GCAGTGAAAGATAAATGATC
DPD-R	GCTATTTCTAGCTCTAAAAC
ARO8-T1-F1	GTAAACCATCCCCATTGAAA
ARO8-T1-R1	TAAGGCTTCACCTCGGTTACAAAAGACTC
ARO8-T2-F2	GTAACCGAGGTGAAGCCTTACTTGAGTTTG
ARO8-T2-R2	ACCTTCAGTCAGTTTCTCAG
For PCR verification	
LJ-YZ-F	GCAGTGAAAGATAAATGATC
LJ-YZ-R	GCTATTTCTAGCTCTAAAAC
ARO8-YZ-F	CCGATCGTTACTATCATGACT
ARO8-YZ-R	CTTCCACTTGCAGTCTTTTAC
For Real-Time qPCR	
ACT1-F	GAAATGCAAACCGCTGCTCA
ACT1-R	TACCGGCAGATTCCAAACCC
ARO8-F	TCACCCAAGCCTCCTTTTCC
ARO8-R	AGGTTGACCAGCACTGAACC
ARO9-F	TGAGCTAGCTGGTGGAATGC
ARO9-R	TGCAATGGGTAGCTCACTGG
ARO10-F	CGGAGACTTCGACGGAAAGT
ARO10-R	GCACTTTAGCTGTTCGGGGA
